# Water-Soluble Fullerene C_60_ Derivatives Are Effective Inhibitors of Influenza Virus Replication

**DOI:** 10.3390/microorganisms11030681

**Published:** 2023-03-07

**Authors:** Ekaterina O. Sinegubova, Olga A. Kraevaya, Aleksandrina S. Volobueva, Alexander V. Zhilenkov, Alexander F. Shestakov, Sergey V. Baykov, Pavel A. Troshin, Vladimir V. Zarubaev

**Affiliations:** 1 Saint Petersburg Pasteur Institute, 14 Ulitsa Mira, 197101 St. Petersburg, Russia; 2 Federal Research Center for Problems of Chemical Physics and Medicinal Chemistry RAS, 1 Prospekt Akademika Semenova, 142432 Chernogolovka, Russia; 3 Faculty of Fundamental Physics & Chemical Engineering, Lomonosov Moscow State University, GSP 1, 1-51 Leninskie Gory, 119991 Moscow, Russia; 4 Institute of Chemistry, Saint Petersburg State University, 7/9 Universitetskaya Nab., 199034 St. Petersburg, Russia; 5 Zhengzhou Research Institute, Harbin Institute of Technology, Longyuan East 7th 26, Jinshui District, Zhengzhou 450003, China; 6 Harbin Institute of Technology, No.92 West Dazhi Street, Nan Gang District, Harbin 150001, China

**Keywords:** influenza virus, hemagglutinin, antiviral agent, fullerene

## Abstract

The influenza virus genome features a very high mutation rate leading to the rapid selection of drug-resistant strains. Due to the emergence of drug-resistant strains, there is a need for the further development of new potent antivirals against influenza with a broad activity spectrum. Thus, the search for a novel, effective broad-spectrum antiviral agent is a top priority of medical science and healthcare systems. In this paper, derivatives based on fullerenes with broad virus inhibiting activities in vitro against a panel of influenza viruses were described. The antiviral properties of water-soluble fullerene derivatives were studied. It was demonstrated that the library of compounds based on fullerenes has cytoprotective activity. Maximum virus-inhibiting activity and minimum toxicity were found with compound **2**, containing residues of salts of 2-amino-3-cyclopropylpropanoic acid (CC_50_ > 300 µg/mL, IC_50_ = 4.73 µg/mL, SI = 64). This study represents the initial stage in a study of fullerenes as anti-influenza drugs. The results of the study lead us conclude that five leading compounds (**1**–**5**) have pharmacological prospects.

## 1. Introduction

Influenza viruses are highly contagious pathogens posing a major threat to human health. Circulating influenza viruses are estimated by the World Health Organization (WHO) to infect 5–10% of the population every year, causing a morbidity rate of 3 to 5 million and mortality rate of 250,000–500,000 deaths annually [1]. Vaccination remains the most effective tool to protect humans against influenza infection. However, vaccination does not always provide complete protection against drifted or, more noticeably, shifted influenza viruses [2]. Antivirals will also play a central role in the treatment and prophylaxis of influenza infections in a pandemic situation, as specific vaccines will take many months to produce. Although there are approved drugs for the treatment of influenza infections, influenza viruses resistant to current antivirals have been reported and continue to emerge. Therefore, there is an urgent need for novel antivirals for the treatment of influenza viral infections in humans.

Three classes of antiviral compounds (M2 inhibitors, neuraminidase inhibitors, viral polymerase complex inhibitors) have been developed and are proven effective in preventing and treating infections caused by susceptible influenza viruses. A range of novel antiviral compounds are under developments that have the advantages of either parenteral delivery, enhanced duration of action, or alternative target(s) and novel mechanisms of activity. Unfortunately, resistance to these agents may develop and become persistent in circulating strains [3]. In the long term, such drug diversity can limit the emergence of influenza virus strains resistant to all available antiviral drugs.

Fullerenes, as well as their derivatives and complexes, are currently being extensively studied. The first fullerene was discovered in 1985 by Harold W. Kroto, Richard E. Smalley, and Robert F. Curl Jr. In 1996, these three scientists were awarded the Nobel Prize for their pioneering efforts [4]. Since their discovery, fullerenes have attracted considerable interest in many fields of research including biomedical applications. Fullerenes, like other nanoparticles, have specific properties, such as small size, large surface area, and high reactivity. These make them interesting for exploitation in the field of technology and medicine. In addition, surface-modified fullerenes may also have increased biological activity due to favorable presentation of active moieties on the particle. 

Available data clearly show that pristine C_60_ has no acute or sub-acute toxicity in a large variety of living organisms, from bacteria and fungi to human leukocytes, drosophilae, mice, rats, and guinea pigs [5]. Fullerenes exhibited very low toxicity after oral exposure and moreover, no evidence of genotoxic or mutagenic potential was observed in vitro. There is limited absorption of pristine fullerenes from the gut, indicating that after repeated exposure no toxicity would be expected. However, functionalized fullerenes with higher solubilities may have different properties in vivo [6,7].

According to biological studies, fullerene derivatives may have the following types of activity: antioxidant [8,9,10], anti-inflammatory [11], antibacterial [12], anticancer [10,13], antiviral [10,14], and others. When searching for drugs, by changing the structure of fullerene derivatives, it is possible to provide an accurate “tuning” of their structures for: obtaining a medicinal substance with a certain biological effect; or for creating means for delivering a medicinal product to the site of exposure without side effects in other tissues or organs.

In this study, the activities of novel, water-soluble fullerene derivatives against influenza viruses were evaluated in vitro. For five fullerene derivatives featuring high selectivity, the mechanisms-of-action and anti-influenza activity spectra were further studied. The obtained results supplement overall understanding of the toxicity and activity of functionalized water-soluble fullerenes.

## 2. Materials and Methods

### 2.1. Cells and Viruses

The following strains from the Saint Petersburg Pasteur Institute viral collection were used in the study: influenza virus A/Aichi/2/68 (H3N2); influenza virus A/mallard/Pennsylvania/1984 (H5N2); influenza virus B/Florida/04/0 6 (Yamagata-like); influenza virus A/Puerto Rico/8/34 (H1N1); influenza virus A/California/07/09 (H1N1)pdm09; influenza virus A/Anhui/1/13 (H7N9); and influenza virus A/Vladivostok/2/09 (H1N1).

Viruses were propagated in the allantoic cavities of 9–11 day old chicken embryos for 48 h at 36 °C. Viral infectious titers were determined in MDCK cells using the end-point titration method. Cells were cultured at 36 °C in a humidified atmosphere with 5% CO_2_. For experiments, cells were seeded in 96-well plates (Thermo Scientific Nunc, Waltham, MA, USA) in the amount of 2 × 10^4^ cells per well in 100 µL of medium. For 24-well plates, 6-well plates, and plastic flasks S = 75 cm^2^ (Thermo Scientific Nunc, Waltham, MA, USA), 2 × 10^6^ cells in 20 mL of medium were used. 

### 2.2. Test Compounds

Libraries of water-soluble fullerene derivatives were synthesized using reactions of chlorofullerene C_60_Cl_6_ with various C-, N-, and S-nucleophiles at the Federal Research Center for Problems of Chemical Physics and Medicinal Chemistry RAS. Compounds were obtained in the form of powders. Weighed portions of these were dissolved in α-MEM medium. 

### 2.3. Culture Medium

Alpha MEM growth medium (Corning, Corning, NY, USA) containing 4 mM L-glutamine, 5% fetal bovine serum FBS (Gibco, Waltham, MA, USA), and penicillin/streptomycin antibiotic (Gibco, Waltham, MA, USA) was used for cell cultivation. For experiments, growth culture medium was replaced with a serum-free maintenance medium with the addition of trypsin (1 μg/mL) for the cultivation of influenza viruses.

### 2.4. Synthesis of Water-Soluble Fullerenes

Compounds **1**–**3** were synthesized from chlorofullerene C_60_Cl_6_ and amino acids using the approach reported earlier [15,16]. Fullerene derivative **4** was obtained by direct arylation of C_60_Cl_6_ with 3-(3-phenylpropanamido)propanoic acid [17]. Reaction of chlorofullerene C_60_Cl_6_ with methyl 2-(thiophen-2-yl)acetate, followed by the treatment with PPh_3_/H_2_O and hydrolysis of the ester groups, allowed us to obtain compound **5**, as described in our recent work [18]. Synthesis and characterization of fullerene derivatives **1** [16], **2** [19], **4** [19], and **5** [18] was reported earlier. Compound **3** was obtained as described in the Supplementary Materials and characterized in the form of a *tert*-butyl ester (**3**-OtBu). Information on the synthesis and characterization of compounds **37** and **39** is also available in the Supplementary Materials. References for the synthesis and characterization of compounds **1**, **2**, **4**–**36**, **38**, and **40**–**45** are provided in Table 1. Oseltamivir carboxylate was used as a reference compound [20,21].

### 2.5. Cytotoxicity Assay

The cytotoxicity of fullerene derivatives was studied using the microtetrazolium test (MTT) [22]. MDCK cells were seeded into 96-well plates for 24 h until a 90% monolayer was formed. Next, cells were incubated with the test substances in a range of concentrations (4–300 μg/mL) for 72 h. The plate was then washed, and 0.1 mL of MTT solution (0.5 μg/mL) was added to each well and incubated for 2 h in 37 °C (humidified atmosphere at 5% CO_2_). Next, the medium was removed, 0.1 mL of dimethyl sulfoxide (DMSO) solution was added to each well, and the resulting formazan was extracted. Optical densities in the wells were measured on a Thermo Multiskan FC spectrophotometer (Thermo Scientific, USA) at a wavelength of 535 nm. The optical density of wells without fullerene derivatives was designated as the negative control (100% viability). Determination of 50% cytotoxic concentration (CC_50_), causing the loss of viability of 50% of cells, was carried out using the GraphPad Prism 7.0. 

### 2.6. CPE Reduction Assay

MDCK cells were seeded into 96-well plates for 24 h until a 90% monolayer was formed. Test substances were added in a range of concentrations (3–300 μg/mL) into the wells of the plates with a cellular monolayer in a volume of 0.1 mL. Plates were incubated for 30 min at 36 °C (5% CO_2_). Next, virus (MOI 0.01) was added to the corresponding wells in maintenance medium with the addition of trypsin and incubated for 1 h. Further, the medium was removed from the wells and replaced with 200 µL of fresh medium with the test compounds at appropriate concentrations. The plates were incubated in 5% CO_2_ at 36 °C for 72 h. Next, cell viability was determined using MTT assay as described above. The 50% inhibitory concentration (IC_50_), causing protection of 50% of infected cells, was calculated using GraphPad Prism 7.0 (USA). Based on the data, the selectivity index (SI) was calculated as the ratio of CC_50_ to IC_50_ for each substance.

### 2.7. Time-of-Addition Experiments

To determine the stage of the influenza viral life cycle that is affected by the compounds, MDCK cells were seeded in 24-well plates and incubated in 5% CO_2_ at 36 °C for 24 h until a confluent monolayer was formed. The cells were washed to remove growth medium. Influenza virus (MOI 0.1) A/Puerto Rico/8/34 (H1N1) was then introduced into the wells. Plates with cells were incubated for 1 h at +4 °C. The virus was then removed, and the plates were washed with clean maintenance medium. Next, at point 0, the substances were introduced into the wells and the plates were placed in a CO_2_ incubator at 37 °C. The compounds were added at the following times relative to the addition of the virus: (–2)–the drug was introduced one hour before the addition of the virus, (–1)–0–simultaneously with the virus, and also 2, 4, and 6 h after infection. The interval (–2)–8 was used as a positive control, where the compounds were present throughout the experiment.

Subsequently, a series of ten-fold dilutions (10^−1^–10^−7^) was prepared from the supernatant and added to 96-well plates with a MDCK cell monolayer. The plates were incubated for 72 h at 37 °C (5% CO_2_). The results of the experiment were ascertained using the hemagglutination assay. For this purpose, 0.1 mL of culture medium was transferred into round-bottom wells and mixed with a 0.1 mL suspension of 1% chicken erythrocytes. The results were checked after 1 h incubation at room temperature. Viral titers were expressed as lgTCID_50_/0.2 mL.

### 2.8. Virus Yield Reduction Assay

MDCK cells were infected with influenza A/Puerto Rico/8/34 (H1N1) virus (MOI of 0.1). Infected cells were incubated at 36 °C for 72 h in a maintenance medium containing antibiotic and trypsin in the presence of the most promising compounds. At the indicated time point (8, 12, 24, 48, 72 h), the culture medium of the infected cells was harvested, and the titer of the progeny virus in the medium was determined by end-point titration, followed by hemagglutination assay, as previously described.

### 2.9. Statistical Analysis

Viral titers were calculated according to the method of Reed and Mench and expressed as log of the 50% infectious dose. Calculation of 50% cytotoxic concentration (CC_50_) and 50% inhibitory concentration (IC_50_) values was performed using GraphPad Prism 7.0 (USA). A 4-parameter equation of the log curve was taken as a working model for analysis (menu items “Nonlinear regression”–“logarithm of the inhibitor–response”). Based on the obtained data, the selectivity index (SI) was calculated for each compound, i.e., the ratio of CC_50_ to IC_50_. Comparison of multiple unrelated samples was performed using the Kruskal–Wallis test (*p* < 0.05).

## 3. Results

### 3.1. Anti-Influenza Activity of Fullerene Derivatives

A total of 45 fullerene derivatives (Table 1), differing in structure of solubilizing addends, were studied. Compounds containing thiophene fragments exhibited antiviral activity but were more toxic than other active compounds. It should be noted that fullerene derivatives wherein the sixth addend is an alkyl group (methyl and ethyl) were less active, and more toxic, than their analogues with a hydrogen atom.

**Table 1:** Antiviral activity of the water-soluble fullerene derivatives against Influenza A/Puerto Rico/8/34 (H1N1) virus in MDCK cells.

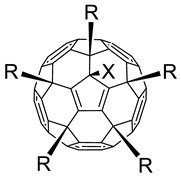						
#	R	X	CC_50_ (µg/mL) ^a^	IC_50_ (µg/mL) ^b^	SI ^c^	Reference for Synthesis and Characterization
1	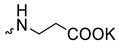	H	>300	6.3 ± 2.5	48	[16,23]
2	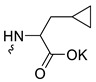	Cl	>300	4.73 ± 2.47	64	[19]
3	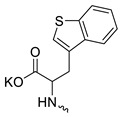	H	>300	5.53 ± 1.72	55	this work
4	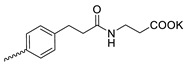	Cl	>300	6.67 ± 2.44	45	[19]
5	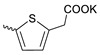	H	33.37 ± 9.42	0.39 ± 0.07	84	[18]
6	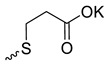	H	>300	106.0 ± 8.54	3	[24]
7	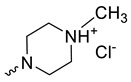	Cl	35.13 ± 1.06	>30	1	[15,25,26]
8	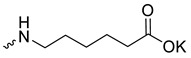	H	14.8 ± 2.10	>11	1	[26]
9	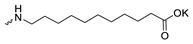	H	>300	>300	1	[26]
10	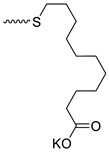	H	>300	31.33 ± 8.66	10	[24]
11	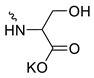	H	>200	34.10 ± 8.91	6	[15,16,23]
12	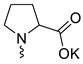	Cl	<3	<3	<1	[23]
13	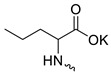	Cl	>300	158.03 ± 13.71	2	[19]
14	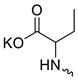	Cl	>300	>300	1	[19]
15	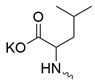	Cl	>300	83.30 ± 7.67	4	[19]
16	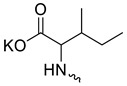	H	>300	40.17 ± 8.26	7	[19]
17	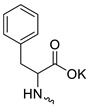	H	200.00 ± 5.00	>100	2	[15,16]
18	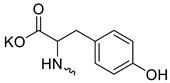	Cl	>200	63.00 ± 10.64	3	[23]
19	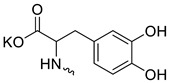	Cl	>300	33.03 ± 9.87	9	[23,27]
20	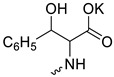	Cl	57.83 ± 6.86	45.07 ± 7.19	1	[23]
21	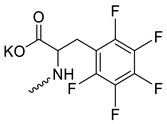	Cl	14.70 ± 2.57	>10	1	[23]
22	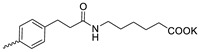	Cl	33.60 ± 6.51	>30	1	[17,19]
23	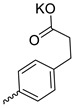	Cl	30.50 ± 4.86	6.93 ± 2.80	4	[28]
24	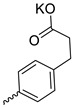	Me	43.60 ± 6.55	>12.5	3	[29]
25	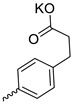	^i^Pr	14.70 ± 3.75	>11	1	[29]
26	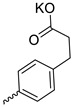	Bu	24.13 ± 4.01	>12.5	2	[29]
27	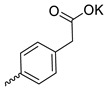	Cl	12.23 ± 3.15	>11	1	[30]
28	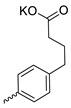	Cl	>300	>300	1	[31]
29	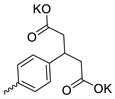	Cl	11.87 ± 4.47	4.00 ± 1.67	3	[29]
30	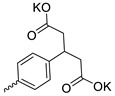	H	9.70 ± 2.40	3.70 ± 1.01	3	[29]
31	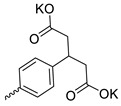	Et	3.67 ± 2.10	>3	1	[29]
32	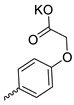	Cl	33.00 ± 9.53	10.33 ± 2.60	3	[28,32]
33	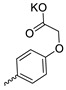	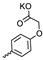	36.60 ± 6.62	1.10 ± 0.26	33	[32]
34	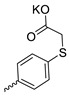	H	32.37 ± 7.07	11.13 ± 1.95	3	[32]
35	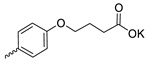	Cl	>300	213.07 ± 13.62	1	[28]
36	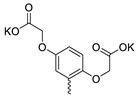	Cl	23.23 ± 7.68	5.47 ± 2.00	4	[33]
37	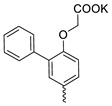	Cl	1.47 ± 0.81	0.24 ± 0.08	6	this work
38	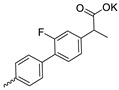	Cl	21.03 ± 7.38	6.97 ± 1.45	3	[17]
39	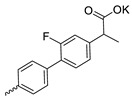	Et	126.07 ± 5.25	96.87 ± 5.85	1	this work
40	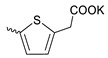	Me	3.70 ± 1.05	>3	1	[18]
41	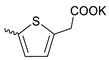	Et	13.80 ± 4.00	>11	1	[18]
42	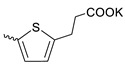	H	11.60 ± 5.60	>11	1	[18]
43	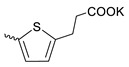	Cl	3.63 ± 1.16	1.03 ± 0.40	4	[18]
44	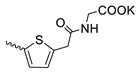	Cl	102.27 ± 10.89	15.83 ± 1.60	6	[17,19]
45	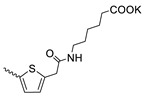	Cl	>300	31.30 ± 5.83	10	[17,19]
Oseltamivir carboxylate			>200 ^d^	0.3 ± 0.02 ^d^	>667	N/A

Notes: ^a^ CC_50_—cytotoxic concentration causing the loss of viability of 50% of cells (µg/mL); ^b^ IC_50_—inhibitory concentration causing a 50% decrease in virus-induced cell death (µg/mL); ^c^ SI—selectivity index, the ratio of CC_50_ to IC_50_; ^d^ data for oseltamivir carboxylate are given in micromoles. The data presented are the mean of three independent experiments. The values for CC_50_ and IC_50_ are presented as mean ± SD.

Compound **5** (Table 1), containing 2-(thiophen-2-yl)acetic acid residues in its structure, occupies first place in terms of SI value (84). However, it has relatively high toxicity (CC_50_ = 33.4 µg/mL). This combination of pharmacological properties cannot be considered optimal. The four remaining leading compounds, which are much less toxic, are also of interest: a fullerene derivative with attached residues of the potassium salt of 3-aminopropanoic acid (**1**); a derivative containing residues of salts of 2-amino-3-cyclopropylpropanoic acid (**2**); a derivative with 2-amino-3-(benzo[b]thiophen-3-yl)propanoic acid (**3**); and a derivative with 3-(3-phenylpropanamido)propanoic acid (**4**). 

The safest (CC_50_ > 300 µg/mL) and most active against the influenza virus was fullerene derivative **2** (containing the residues of salts of 2-amino-3-cyclopropylpropanoic acid). Compound **38** (with five attached residues of potassium salt of 2-([1,1′-biphenyl]-2-yloxy)acetic acid and a chlorine atom) combines the properties of the most active and toxic functionalized fullerene derivative (CC_50_ = 1.47 µg/mL, IC_50_ = 0.24 µg/mL), while having low SI = 6.

**Figure 1:**
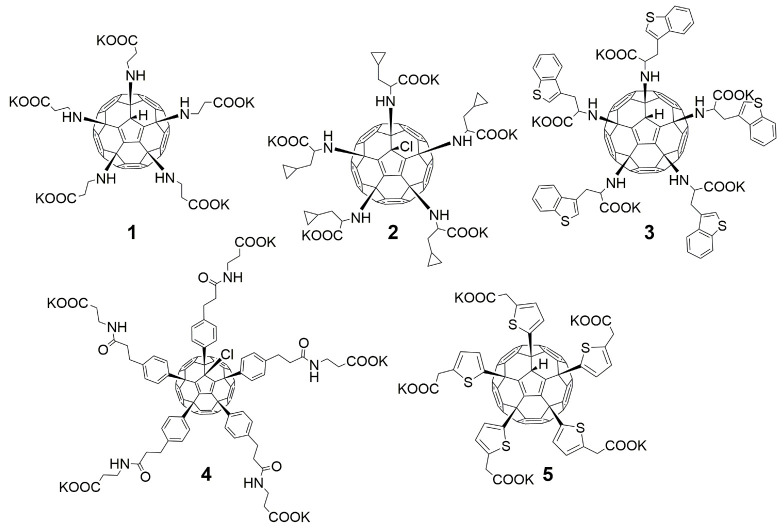
Molecular structures of water-soluble fullerene derivatives **1**–**5**.

Taking into account that C_60_ fullerene itself has low toxicity, the obtained data on low CC_50_ values (<300 μg/mL) for more than 64.4% of derivatives (29 out of 45) are due to the cytotoxic activity of addends. A total of 4 out of the 45 studied compounds (17.7%) had a pronounced antiviral effect and minimal toxicity, among which 5 leading compounds (**1**–**5**, Figure 1) were found to have the maximum inhibitory effect against the influenza virus (Table 1). The highest activity and minimal toxicity (CC_50_ > 300 µg/mL, IC_50_ = 4.73 µg/mL, SI = 64) was demonstrated by compound **2** (containing residues of salts of 2-amino-3-cyclopropylpropanoicacid). 

**Figure 2:**
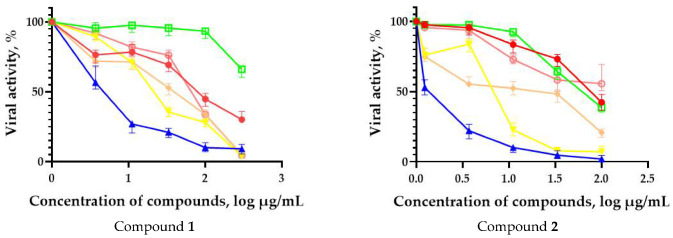

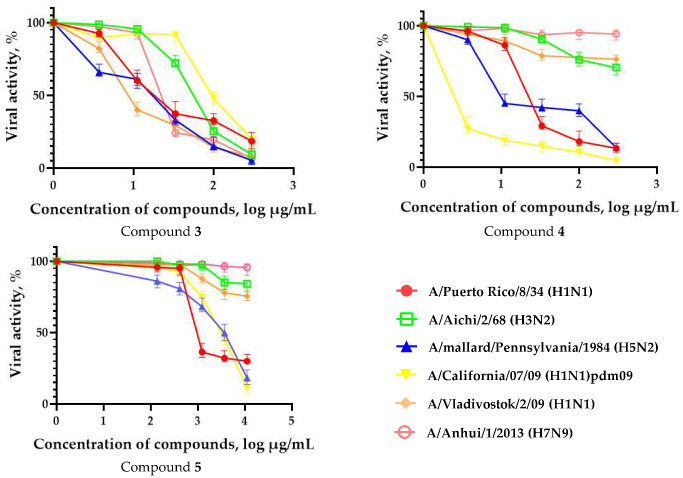
Spectrum of antiviral activity of leading fullerene derivatives (**1**–**5**). MDCK cells were infected with influenza viruses (MOI 0.01) and cultivated in the presence of compounds for 72 h followed by CPE reduction test. Figures represent 4-parameter log curves plotted using GraphPad Prism 6.01 for different subtypes of influenza virus. The values of viral activity (dots) are presented as mean ± SD of three independent experiments.

In order to evaluate the activity spectrum of test compounds, we studied their virus-inhibiting properties against a panel of influenza virus subtypes of different origin in vitro. The results are presented in Figure 2. The most promising compounds demonstrated a pronounced inhibitory effect against different influenza virus subtypes. 

The fullerene derivative with attached residues of the potassium salt of 3-aminopropanoic acid (1) suppressed the replication of influenza viruses of strains A/Puerto Rico/8/34 (H1N1) and A/California/07/09 (H1N1)pdm09. At the same time, compounds containing residues of salts of 2-amino-3-cyclopropylpropanoic (**2**), 2-amino-3-(benzo[b]thiophen-3-yl)propanoic (**3**), and 3-(3- phenylpropanamido)propanoic acids (**4**) in their structure were also active against the oseltamivir-resistant A/Vladivostok/2/09 (H1N1) strain and the A/mallard/Pennsylvania/1984 (H5N2) avian influenza strain.

### 3.2. Time-of-Addition Experiments

**Figure 3:**
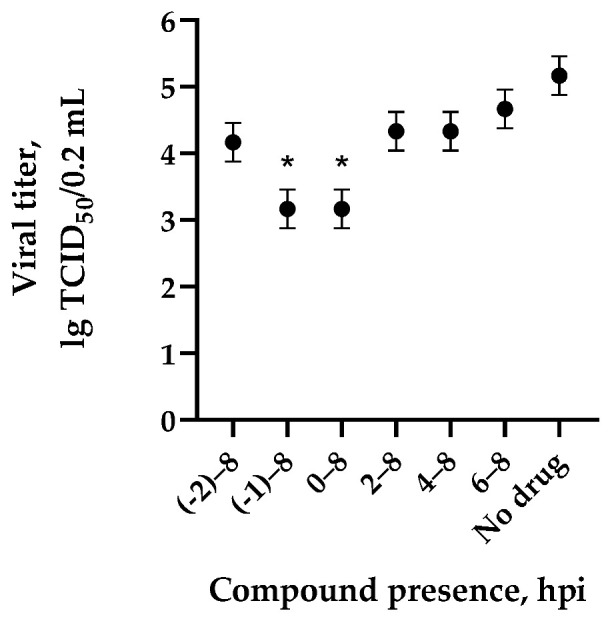
The effect of compound **1** on the values of infectious activity (Me [95% CI]) of the influenza A/Puerto Rico/8/34 (H1N1) virus depending on the time of addition to MDCK cell culture. The starting point of incubation was referred as 0. Cells were treated with fullerene derivatives for the time periods as following: (-2)–(-8) (before infection); (-1)–8 (simultaneously to absorption); 0–8; 2–8; 4–8; 6–8 and (-2)–8 hpi. Data are presented as mean ± SD of three independent experiments. *—differences are significant relative to “no drug” (Kruskal–Wallis test, *p* < 0.05).

The mechanisms of inhibitory action of the lead compounds **1**–**5** (Figure 1) were studied in time-of-addition tests. Infected cells were incubated with leader compounds at different stages of the viral replication cycle. To determine the stage of the viral life cycle at which the leader compounds exhibit maximum antiviral activity, test substances were added to the infected cell culture at different time points relative to the moment of infection. Then, after passing through one viral cycle (8 h), the infectious activity of viral progeny was evaluated. The results of the experiment are shown in Figure 3 using compound **1** as an example. Herafter, ‘hours post infection’ will be abbreviated (hpi).

The life cycle of the influenza virus consists of seven stages, in which strictly defined viral components play a key role in the replicative process. Direct-acting drugs can display activity at specific stages of the viral life cycle, thus inhibiting the entire replicative cycle. Consequently, determination of the stage of the influenza virus life cycle affected by the studied compounds allows us to draw a conclusion about the drug’s viral target. In accordance with the results, the viral titer in the control was 10^5^ TCID_50_/0.2 mL. The presence of compound **1** at the early stages of the viral life cycle (with simultaneous incubation of cells with the virus (–1) for 8 hpi, in the first hours of the viral life cycle for 0–8 hpi) led to a decrease in the infectious activity of viral progeny. The inhibitory effect of the leader compounds weakened when they were added at later stages of the viral cycle. The obtained temporal characteristic (of leading compound activity) suggests that the mechanism of action of new fullerene derivatives is based on inhibition of the early stages of viral replication, including the process of virus adsorption onto cells upon contact of the drug with extracellular virions.

### 3.3. One-Step Growth Curve in the Presence of the Most Active Compounds ** *1***–** *5***

**Figure 4:**
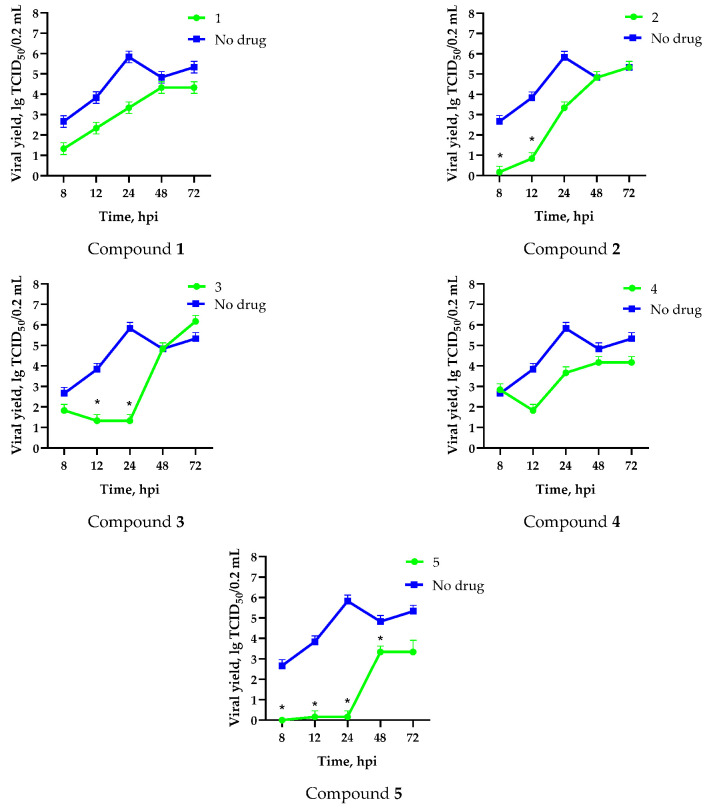
Effect of compounds **1**–**5** on influenza A/Puerto Rico/8/34 (H1N1) viral growth in MDCK cells. Cells were infected with virus (MOI 0.1) in the presence of compounds. Aliquots of culture medium were taken at 8, 12, 24, and 48 hpi. Titration of viral progeny by end-point dilution in MDCK cells with virus detection by hemagglutination assay was then performed. Data are presented as the mean ± SD of three independent experiments. *—differences are significant relative to “no drug” (Kruskal–Wallis test, *p* < 0.05).

In order to characterize the mechanism of antiviral action of the fullerene derivatives on the replication of the influenza A/Puerto Rico/8/34 (H1N1) virus, a one-step growth curve was examined in the presence of the test compounds. In the absence of the test substances, the infectious titer of the virus was 3 lg TCID_50_/0.2 mL after just 8 hpi, reaching a plateau at 24 hpi (Figure 4). The addition of water-soluble fullerene derivatives at the beginning of viral replication caused a notable decrease in the number of infectious viruses produced during this period, followed by restoration of infectious virus formation from 12 to 48 hpi. This was likely due to non-specific decomposition or metabolic degradation of the compounds over time. 

## 4. Discussion

Over the past few years, many research groups have begun to investigate biological application of fullerenes. The fullerene molecule is highly active due to a large amount of double bonds that can react with various radicals, including biologically active ones. It shows high and, in some cases, even unique antioxidant properties. Fullerene can penetrate cell membranes, modulate ion transport, cross the blood–brain barrier, and has adhesive potential. These properties can be used for synthesis of new immunomodulators, adjuvants, and vaccine holders, for example for HIV-antigens. As reported earlier, water-soluble fullerene adducts have photodynamic, antibacterial, as well as pro- and antioxidant capacity. They can be used as scaffolds for the synthesis of anticancer, neuroprotective, and antiviral drugs [10,34]. We have previously reported on high antiherpetic activity with water-soluble fullerene derivatives [18].

The antiviral properties of novel, water-soluble fullerene derivatives, and their mechanism-of-action against influenza were studied in vitro. It was shown that compounds from the library of water-soluble fullerenes have cytoprotective activity against the influenza virus. The maximum virus-inhibiting activity and minimum toxicity was shown by the derivative with attached residues of the potassium salt of β-alanine. In the present work, we observed a wide range of activity of water-soluble fullerene derivatives against influenza viruses of differing antigenic subtype. The mechanism of antiviral activity of the most promising compounds is the inhibition of early stages of viral replication (0–2 hpi). 

The main issue for biomedical application of fullerenes concerns insolubility in water and aqueous solutions [34]. The current search for the most active fullerene derivative is promising, especially taking into account the possibilities of increasing the bioavailability of a potential drug using liposomal drug delivery complexes [35]. Hydroxylation is one of the cheapest and easiest ways to dissolve fullerenes in water and does not require extensive purification of the resultant product. Many studies are currently focused on non-functionalized (pristine) fullerenes in aqueous dispersions since they do not participate in metabolic processes due to their open surface [36,37,38,39]. However, even slight surface derivatization can increase the activity of fullerenes. In our work, fullerenes with solubilizing addends were studied. This certainly increases their bioavailability and potential medicinal application accordingly.

Thus, this research represents the initial stage in a study of water-soluble fullerene derivatives as anti-influenza drugs. It allows us to conclude that five leading compounds (**1**–**5**, Figure 1) have pharmacological prospects. These data are a key advancement that could be used in future strategies to refine fullerene-based drug design. Our analysis provides valuable new information for the design of novel, anti-influenza drugs. We believe the information presented here will be beneficial for the future development of novel fullerene-based drugs against influenza A.

An important issue in the search for antiviral drugs is assessment of the risk of drug resistance development. Further study should evaluate the ability of water-soluble fullerene derivatives to develop viral resistance, potentially leading to the emergence of strains with reduced drug susceptibility. Consequently, amino acid substitutions in the influenza virus proteins which lead to drug resistance should be mapped. The data obtained in this paper serve as a basis for continuing research on water-soluble fullerene derivatives as potential anti-influenza drugs.

## Data Availability

The data that support the findings of this study are available from the corresponding author, E.O.S., upon reasonable request.

## Supplementary Materials

The following supporting information can be downloaded at: https://www.mdpi.com/article/10.3390/microorganisms11030681/s1.
