# Supplementary material for: Water-Soluble Fullerene C60 Derivatives Are Effective Inhibitors of Influenza Virus Replication
Source: Microorganisms. 2023 Mar 7;11(3):681. doi: 10.3390/microorganisms11030681 (PMC10053623; doi:10.3390/microorganisms11030681)
Supplement: Supplementary file 1 [file microorganisms-11-00681-s001.zip › microorganisms-2251317-supplementary.pdf]

Supporting Information for

Water-soluble fullerene C_{60} derivatives are effective inhibitors of influenza virus reproduction

Ekaterina O. Sinegubova [1,*], Olga A. Kraevaya [2], Aleksandrina S. Volobueva [1], Alexander V. Zhilenkov [2], Alexander F. Shestakov [2,3], Sergey V. Baykov [4,*], Pavel A. Troshin [5,6,2], Vladimir V. Zarubaev [1]

[1] St. Petersburg Pasteur Institute, 14 Mira st., St. Petersburg 197101, Russia

[2] Federal Research Center for Problems of Chemical Physics and Medicinal Chemistry RAS, Semenov ave., 1, Chernogolovka 142432, Russia

[3] Faculty of Fundamental Physics & Chemical Engineering, Lomonosov Moscow State University, GSP 1, 1-51 Leninskie Gory, Moscow 119991, Russia

[4] Institute of Chemistry, Saint-Petersburg State University, 7/9 Universitetskaya Nab., St. Petersburg 199034, Russia

[5] Zhengzhou Research Institute, Harbin Institute of Technology, Longyuan East 7th 26, Jinshui District, 450003 Zhengzhou, Henan Province, China

[6] Harbin Institute of Technology, No.92 West Dazhi Street, Nan Gang District, 150001 Harbin, China

* Correspondence: sinek489@gmail.com (E.O.S.), s.baykov@spbu.ru (S.V.B.)

Compound **3.** Method of synthesis is similar to the synthesis of compound **9** from [1] yield 47%.

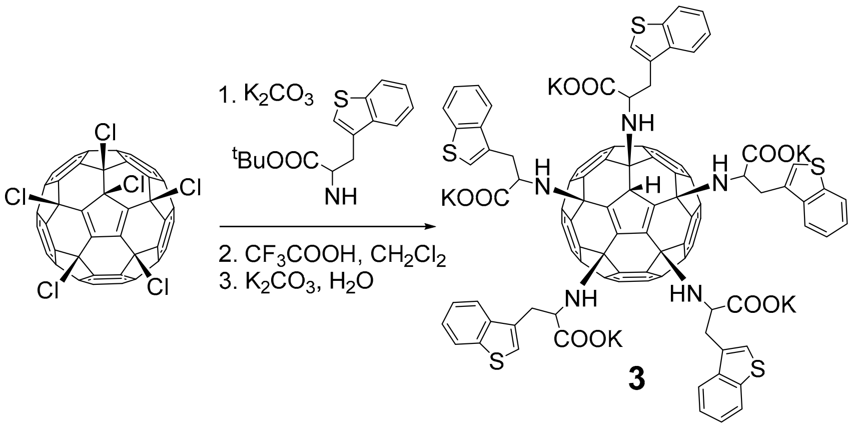

Compound **3**–O^{t}Bu. ^1H NMR (500 MHz, $CDCl_3$, δ, ppm): 8.12–7.29 (m, 20H), 7.25–7.03 (m, 5H), 4.30–3.22 (m, 15H), 1.50–0.98 (m, 45H). ^{13}C NMR (126 MHz, $CDCl_3$, δ, ppm): 167.42, 153.62, 151.68, 149.31, 149.14, 148.60, 148.55, 148.49, 148.00, 147.98, 147.68, 147.35, 147.27, 147.11, 145.18, 144.95, 144.49, 144.11, 143.60, 143.32, 142.83, 142.59, 142.37, 141.79, 141.30, 140.89, 140.43, 138.31, 136.07, 129.05, 128.24, 126.20, 125.31, 124.50, 124.45, 124.43, 124.33, 124.15, 122.99, 122.87, 122.71, 121.85, 84.55, 58.00, 56.51, 56.06, 55.96, 55.66, 53.49, 29.71, 29.38, 27.85, 27.71, 27.47.

Compound **37-OMe**. Method of synthesis is similar to compound **1a** from [2] yield: 41%.

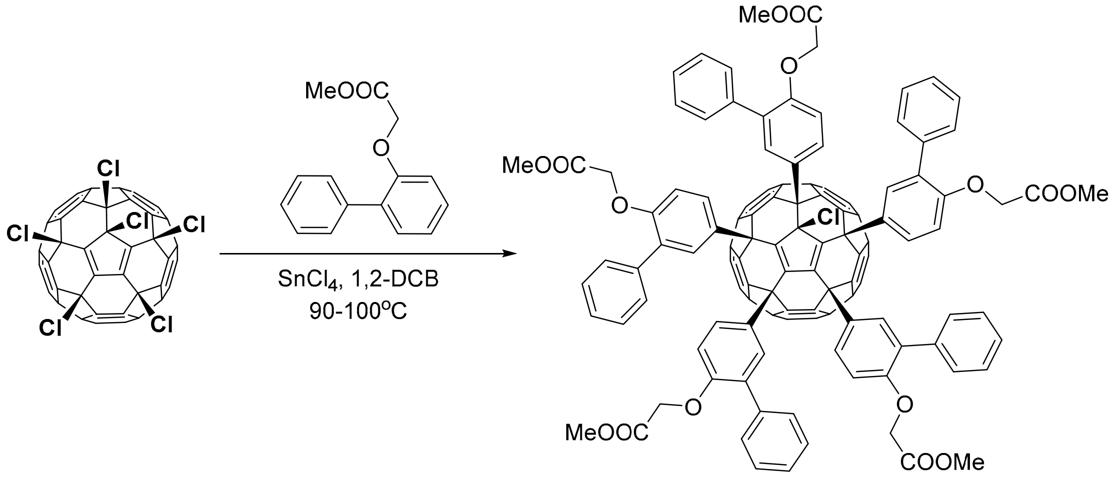

^1H NMR (500 MHz, $CDCl_3$, δ, ppm): 8.08-7.19 (m, 36H), 6.80–6.51 (m, 4H), 4.61 (s, 2H, OC<u>H</u>2), 4.59 (s, 2H, OC<u>H</u>2), 4.57 (s, 2H, OC<u>H</u>2), 4.50 (s, 2H, OC<u>H</u>2), 4.47 (s, 2H, OC<u>H</u>2), 3.83 (s, 3H, OC<u>H</u>3), 3.81 (s, 6H, OC<u>H</u>3), 3.80 (s, 3H, OC<u>H</u>3), 3.78 (s, 3H, OC<u>H</u>3). ^{13}C NMR (126 MHz, $CDCl_3$, δ, ppm): 169.27 (<u>C</u>OOCH3), 169.23 (<u>C</u>OOCH3), 169.22 (<u>C</u>OOCH3), 169.13 (<u>C</u>OOCH3), 157.23,

155.80, 154.52, 154.32, 154.28, 154.21, 154.13, 153.95, 153.09, 151.94, 150.90, 150.82, 149.90, 148.84, 148.80, 148.74, 148.66, 148.49, 148.47, 148.33, 148.29, 148.20, 147.93, 147.82, 147.43, 147.39, 147.27, 147.26, 147.06, 146.44, 145.65, 145.43, 145.34, 144.87, 144.68, 144.63, 144.55, 144.51, 144.46, 144.38, 144.35, 144.32, 144.29, 144.24, 144.23, 143.89, 143.62, 143.38, 143.27, 143.15, 143.12, 142.87, 142.77, 137.57, 137.50, 137.41, 137.34, 137.29, 137.25, 134.13, 132.44, 132.14, 131.84, 131.82, 131.74, 131.55, 131.51, 131.29, 130.92, 130.87, 130.38, 130.08, 129.78, 129.64, 129.53, 129.45, 129.16, 128.60, 128.54, 128.29, 128.12, 128.00, 127.95, 127.91, 127.17, 127.15, 127.13, 127.02, 112.76, 112.62, 112.55, 111.32, 77.23 ($\underline{C}_{sp^3\,cage}$-Cl), 65.77 ($\underline{C}H_2$), 65.66 ($\underline{C}H_2$), 65.55 ($\underline{C}H_2$), 65.49 ($\underline{C}H_2$), 65.42 ($\underline{C}H_2$), 62.88 ($\underline{C}_{sp^3\,cage}$), 60.29 ($\underline{C}_{sp^3\,cage}$), 60.15 ($\underline{C}_{sp^3\,cage}$), 57.62 ($\underline{C}_{sp^3\,cage}$), 57.53 ($\underline{C}_{sp^3\,cage}$), 52.28 (O$\underline{C}H_3$), 52.24 (O$\underline{C}H_3$), 52.20 (O$\underline{C}H_3$), 52.19 (O$\underline{C}H_3$),

HRMS (MALDI TOF, DCTB matrix): found m/z = 1926.45; calcd for $C_{135}H_{65}O_{15}$ ($[M–Cl]^-$) m/z = 1926.44.

Compound **37-OH.** Method of synthesis is similar to compound **A1a** from [2] yield: 96%.

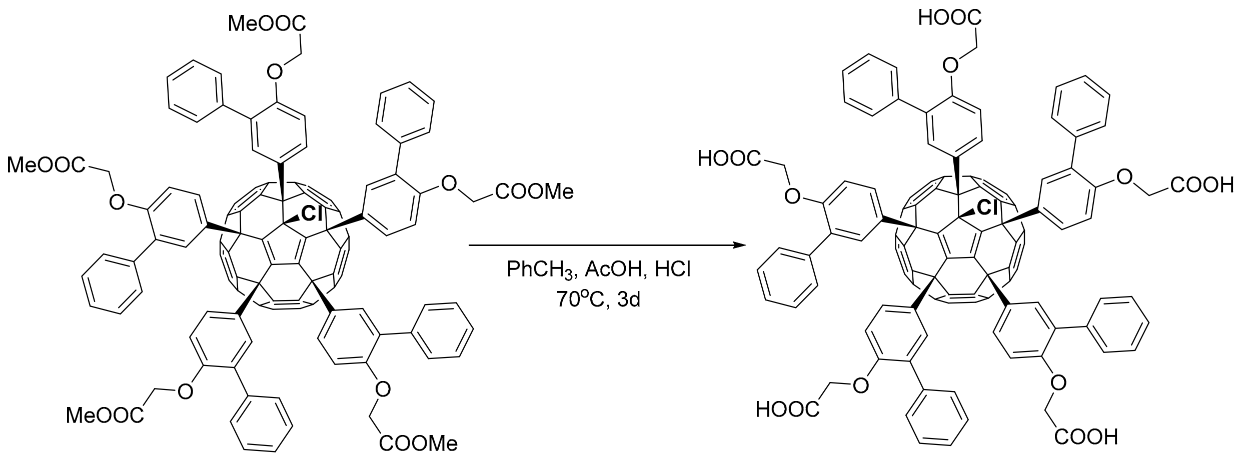

37-OMe **37-OH**

Compound **37-OH.** ^{1}H NMR (500 MHz, $(CD_3)_2SO$, δ, ppm): 7.96–7.35 (m, 10H), 7.34–6.57 (m, 30H), 4.72 (s, 2H), 4.69 (s, 2H), 4.66 (s, 2H), 4.58 (s, 2H), 4.54 (s, 2H). ^{13}C NMR (126 MHz, $(CD_3)_2SO$, δ, ppm): 170.47 ($\underline{C}OOH$), 170.43 ($\underline{C}OOH$), 170.29 ($\underline{C}OOH$), 170.26 ($\underline{C}OOH$), 157.13, 156.23, 155.00, 154.79, 154.74, 154.67, 154.60, 154.37, 152.90, 151.72, 151.10, 150.85, 150.21, 148.67, 148.58, 148.54, 148.46, 148.33, 148.14, 148.08, 148.04, 147.78, 147.68, 147.29, 147.25, 147.15, 147.11, 146.98, 146.80, 145.53, 145.47, 145.12, 145.02, 144.48, 144.43, 144.31, 144.25, 144.20, 144.16, 144.13, 143.76, 143.59, 143.54, 143.31, 143.00, 142.97, 142.70, 142.68, 137.79, 137.66, 137.54, 137.46, 137.31, 136.49, 131.08, 131.04, 131.01, 130.95, 130.38, 130.17, 129.73, 129.65, 129.54, 129.43, 129.35, 128.94, 128.48, 128.37, 128.29, 127.43, 127.38, 113.64, 113.40, 113.15, 112.95, 77.52 ($\underline{C}_{sp^3\,cage}$-Cl), 65.48 ($\underline{C}H_2$), 65.29 ($\underline{C}H_2$), 65.22 ($\underline{C}H_2$), 65.18 ($\underline{C}H_2$), 65.01 ($\underline{C}H_2$), 62.83 ($\underline{C}_{sp^3\,cage}$), 60.44 ($\underline{C}_{sp^3\,cage}$), 60.04 ($\underline{C}_{sp^3\,cage}$), 57.53 ($\underline{C}_{sp^3\,cage}$), 57.33 ($\underline{C}_{sp^3\,cage}$).

Compound **37.** Method of synthesis is similar to compound **K1a** from [2] yield: 93%.

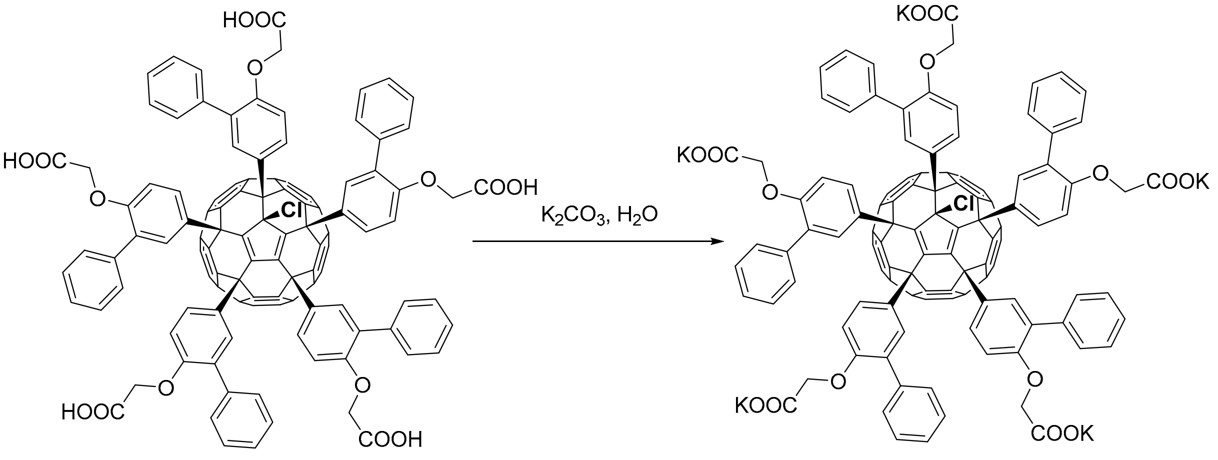

37-OH **37**

Compound **39-OMe.** Method of synthesis is similar to compound **1d** from [2] yield: 56%.

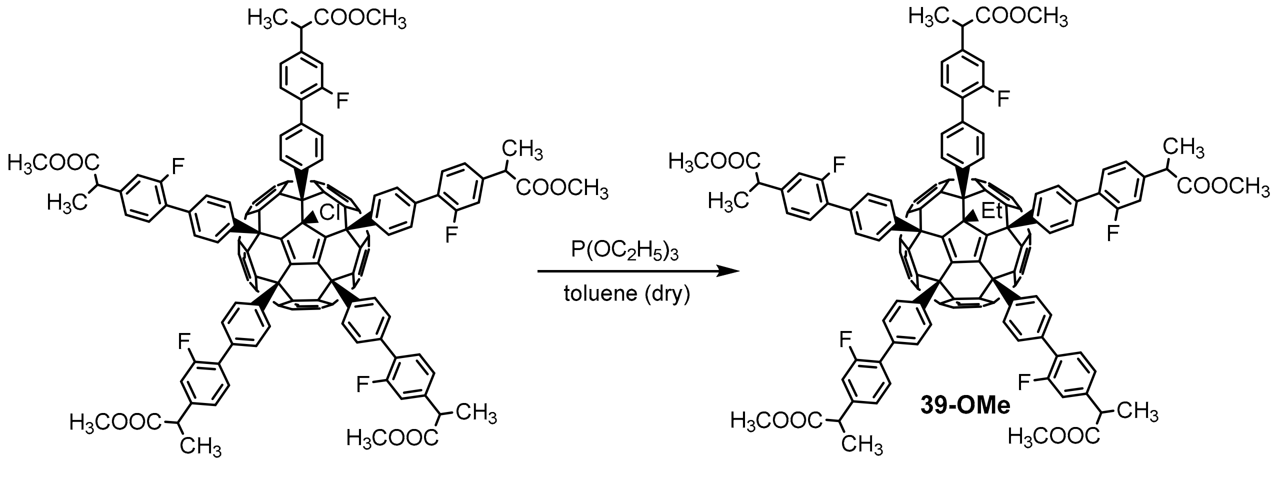

39-OMe

Compound **39-OMe**. ^{1}H NMR (600 MHz, $CDCl_3$, δ, ppm): 7.96 (d, J = 8.3 Hz, 4H, $C\underline{H}_{aromatic}$), 7.84 (d, J = 8.3 Hz, 4H, $C\underline{H}_{aromatic}$), 7.56 (d, J = 7.9 Hz, 8H, $C\underline{H}_{aromatic}$), 7.51–7.41 (m, 6H, $C\underline{H}_{aromatic}$), 7.39–7.31 (m, 3H, $C\underline{H}_{aromatic}$), 7.23–7.04 (m, 10H, $C\underline{H}_{aromatic}$), 3.83–3.75 (m, 5H, $C\underline{H}(CH_3)$), 3.73 (s, 6H, $OC\underline{H}_3$), 3.72 (s, 6H, $OC\underline{H}_3$), 3.69 (s, 3H, $OC\underline{H}_3$), 1.68 (q, J = 7.1 Hz, 2H, $C\underline{H}_2CH_3$), 1.57 (d, J = 7.2 Hz, 6H, $CH(C\underline{H}_3)$), 1.55 (d, J = 7.2 Hz, 6H, $CH(C\underline{H}_3)$), 1.51 (d, J = 7.2 Hz, 3H, $CH(C\underline{H}_3)$), 1.07 (t, J = 7.1 Hz, 3H, $CH_2C\underline{H}_3$). ^{13}C NMR (151 MHz, $CDCl_3$, δ, ppm): 174.47 ($\underline{C}OOCH_3$), 174.44 ($\underline{C}OOCH_3$), 174.40 ($\underline{C}OOCH_3$), 160.62, 160.56, 160.45, 158.97, 158.91, 158.81, 156.95, 155.93, 153.26, 151.34, 148.90, 148.83, 148.69, 148.56, 148.40, 148.31, 148.18, 147.97, 147.57, 147.44, 147.37, 147.16, 145.60, 145.34, 144.81, 144.77, 144.50, 144.45, 144.27, 144.17, 144.08, 143.90, 143.61, 142.81, 142.33, 142.14, 142.12, 142.09, 142.07, 142.04, 141.99, 139.84, 138.29, 135.13, 135.04, 134.21, 130.87, 130.80, 130.77, 130.74, 130.70, 129.49,

129.48, 129.21, 129.19, 129.05, 128.54, 128.52, 128.04, 127.21, 127.12, 127.02, 126.93, 126.81, 126.72, 123.70, 123.68, 123.67, 123.65, 123.58, 123.56, 115.48, 115.45, 115.32, 115.29, 115.16, 65.76 ($\underline{C}_{sp^3\,cage}$), 63.43 ($\underline{C}_{sp^3\,cage}$), 61.07 ($\underline{C}_{sp^3\,cage}$), 58.58 ($\underline{C}_{sp^3\,cage}$), 52.32 ($O\underline{C}H_3$), 52.30 ($O\underline{C}H_3$), 52.26 ($O\underline{C}H_3$), 44.96 ($\underline{C}H$), 44.94 ($\underline{C}H$), 44.87 ($\underline{C}H$), 33.83 ($\underline{C}H_2CH_3$), 18.49 ($CH(\underline{C}H_3)$), 18.46 ($CH(\underline{C}H_3)$), 18.40 ($CH(\underline{C}H_3)$), 9.90 ($CH_2\underline{C}H_3$).

HRMS (MALDI TOF, DCTB matrix): found m/z = 2035.68; calcd for $C_{142}H_{75}F_5O_{10}$ ($[M]^-$) m/z = 2035.53.

Compound **39-OH**. Method of synthesis is similar to compound **A1d** from [2] yield: 95%.

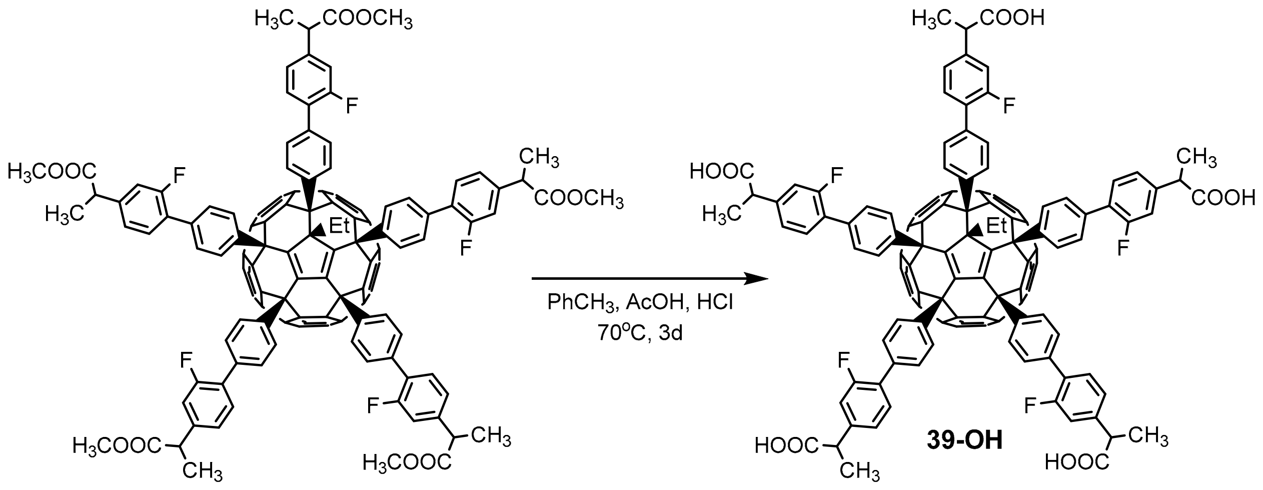

Compound **39-OH**. ^{1}H NMR (600 MHz, $(CD_3)_2SO$, δ, ppm): 12.45 (br s, 5H, $COO\underline{H}$), 7.92 (d, J = 7.5 Hz, 4H, $C\underline{H}_{aromatic}$), 7.78 (d, J = 7.4 Hz, 4H, $C\underline{H}_{aromatic}$), 7.63–7.30 (m, 16H, $C\underline{H}_{aromatic}$), 7.28–7.03 (m, 11H, $C\underline{H}_{aromatic}$), 3.79–3.65 (m, 5H, $C\underline{H}(CH_3)$), 1.62–1.54 (m, 2H, $C\underline{H}_2CH_3$), 1.39 (d, J = 7.2 Hz, 6H, $CH(C\underline{H}_3)$), 1.37 (d, J = 7.2 Hz, 6H, $CH(C\underline{H}_3)$), 1.32 (d, J = 6.9 Hz, 3H, $CH(C\underline{H}_3)$), 1.06–0.97 (m, 3H, $CH_2C\underline{H}_3$). ^{13}C NMR (151 MHz, $(CD_3)_2SO$, δ, ppm): 175.28 ($\underline{C}OOH$), 175.26 ($\underline{C}OOH$), 175.21 ($\underline{C}OOH$), 160.25, 160.20, 158.61, 158.56, 158.47, 156.90, 156.41, 153.35, 151.46, 148.61, 148.59, 148.33, 148.16, 148.07, 147.93, 147.76, 147.62, 147.21, 147.17, 146.94, 146.76, 146.53, 146.31, 146.02, 145.58, 145.29, 144.53, 144.42, 144.33, 144.08, 143.96, 143.90, 143.81, 143.62, 142.64, 142.10, 139.38, 137.77, 137.74, 135.20, 131.06, 130.87, 129.94, 129.69, 128.93, 128.18, 128.15, 126.14, 125.94, 124.55, 124.52, 118.52, 115.81, 115.76, 115.65, 115.61, 65.69 ($\underline{C}_{sp^3\,cage}$), 63.33 ($\underline{C}_{sp^3\,cage}$), 61.05 ($\underline{C}_{sp^3\,cage}$), 58.46 ($\underline{C}_{sp^3\,cage}$), 44.60 ($\underline{C}H$), 44.57 ($\underline{C}H$), 44.49 ($\underline{C}H$), 34.08 ($\underline{C}H_2CH_3$), 18.74 ($CH(\underline{C}H_3)$), 18.69 ($CH(\underline{C}H_3)$), 10.04 ($CH_2\underline{C}H_3$).

Compound **39**. Method of synthesis is similar to compound **K1d** from [2] yield: 98%.

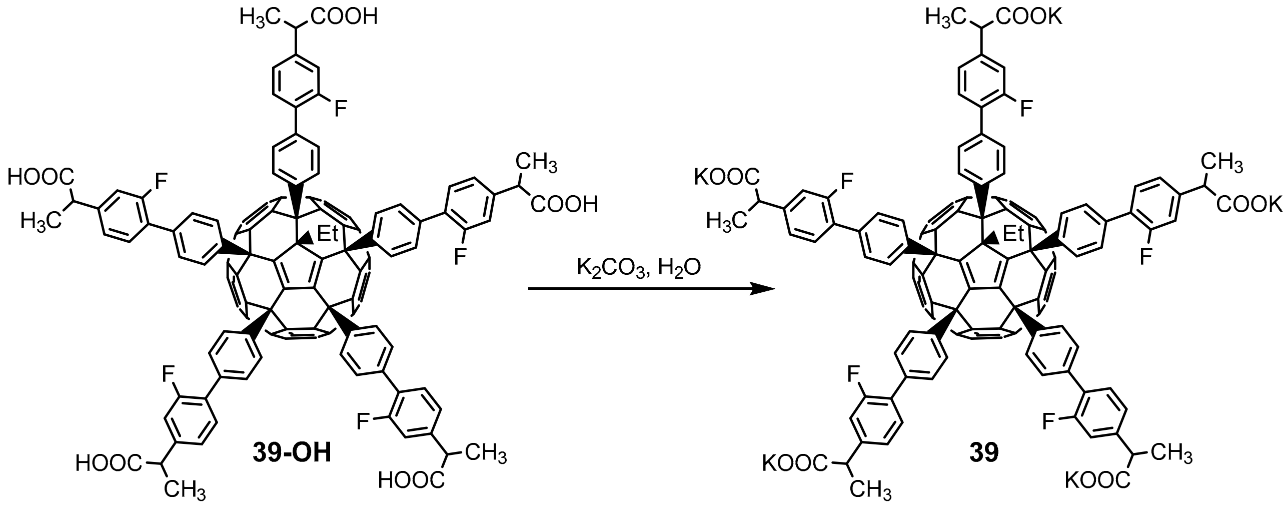

References

1. Hsieh, F.-Y., Zhilenkov, A.V., Voronov, I.I., Khakina, E.A., Mischenko, D.V., Troshin, P.A., Hsu, S.-H. Water-Soluble Fullerene Derivatives as Brain Medicine: Surface Chemistry Determines If They Are Neuroprotective and Antitumor. *ACS Appl. Mater. Interfaces* **2017**, *9*, 11482–11492. https://doi.org/10.1021/acsami.7b01077
2. Kraevaya, O.A., Peregudov, A.S., Fedorova, N.E., Klimova, R.R., Godovikov, I.A., Mishchenko, D.V., Shestakov, A.F., Schols, D., Kushch, A.A., Troshin, P.A. Thiophene-based water-soluble fullerene derivatives as highly potent antiherpetic pharmaceuticals. *Org. Biomol. Chem.* **2020,** *18*, 8702–8708. https://doi.org/10.1039/D0OB01826K
